# Addition of Clofazimine Enhances the Activity of Standard Treatment Regimen in a Mouse Model of Tuberculous Meningitis

**DOI:** 10.1093/infdis/jiag123

**Published:** 2026-08-03

**Authors:** Xueyi Chen, Carlos E. Ruiz-Gonzalez, Yuderleys Masias-Leon, Medha Singh, Charles A. Peloquin, Sanjay K. Jain

**Affiliations:** 1 Department of Pediatrics, Johns Hopkins University School of Medicine, Baltimore, Maryland, USA; 2 Center for Tuberculosis Research, Johns Hopkins University School of Medicine, Baltimore, Maryland, USA; 3 Department of Pediatrics, Cincinnati Children’s Hospital Medical Center, Cincinnati, Ohio, USA; 4 Cincinnati Children’s Center for Molecular Imaging and Precision Medicine, Cincinnati Children’s Hospital Medical Center, Cincinnati, Ohio, USA; 5 Infectious Disease Pharmacokinetics Laboratory, Pharmacotherapy and Translational Research, University of Florida College of Pharmacy, Gainesville, Florida, USA

**Keywords:** tuberculosis, tuberculous meningitis, chemotherapy, clofazimine

## Abstract

Tuberculous meningitis causes substantial mortality due to limited penetration of antibiotics to the central nervous system and excessive neuroinflammation. This study evaluated clofazimine as an adjunct to the standard first-line tuberculosis regimen, in a mouse model. Mice were infected intracerebrally with *Mycobacterium tuberculosis* and treated for 6 weeks. Addition of clofazimine markedly reduced the brain bacterial burden, achieving measurable brain tissue concentrations despite minimal cerebrospinal fluid penetration, as well as attenuated brain tissue inflammation. These findings indicate that clofazimine enhances bactericidal activity of the standard regimen and mitigate neuroinflammation, supporting further investigation and clinical evaluation for tuberculous meningitis.

Tuberculous meningitis (TB meningitis), the most severe form of tuberculosis (TB), remains associated with high mortality and neurological sequelae despite treatments with existing regimens [1]. Poor outcomes stem largely from inadequate antibiotic penetration into the central nervous system (CNS) and intense neuroinflammation that causes brain injury. New therapeutic agents with adequate intracerebral drug exposure, along with strategies to mitigate inflammation are urgently needed.

Clofazimine, a riminophenazine compound with both anti-mycobacterial and immunomodulatory properties (eg, enhancement of anti-PD-1 immunotherapies), has gained renewed interest as an adjunct agent in multidrug-resistant TB regimens. Its favorable oral bioavailability, long half-life, and tendency to accumulate in tissues raise the possibility that it may be effective in CNS disease, although data on its brain penetration and therapeutic role in TB meningitis remain limited [2].

This study evaluates the addition of clofazimine to the standard, first-line TB regimen in a mouse model of TB meningitis, assessing its effects on intracerebral bacterial clearance, tissue antibiotic concentrations, and neuroinflammatory responses.

## METHODS

### Animal Infection

**Figure 1.**
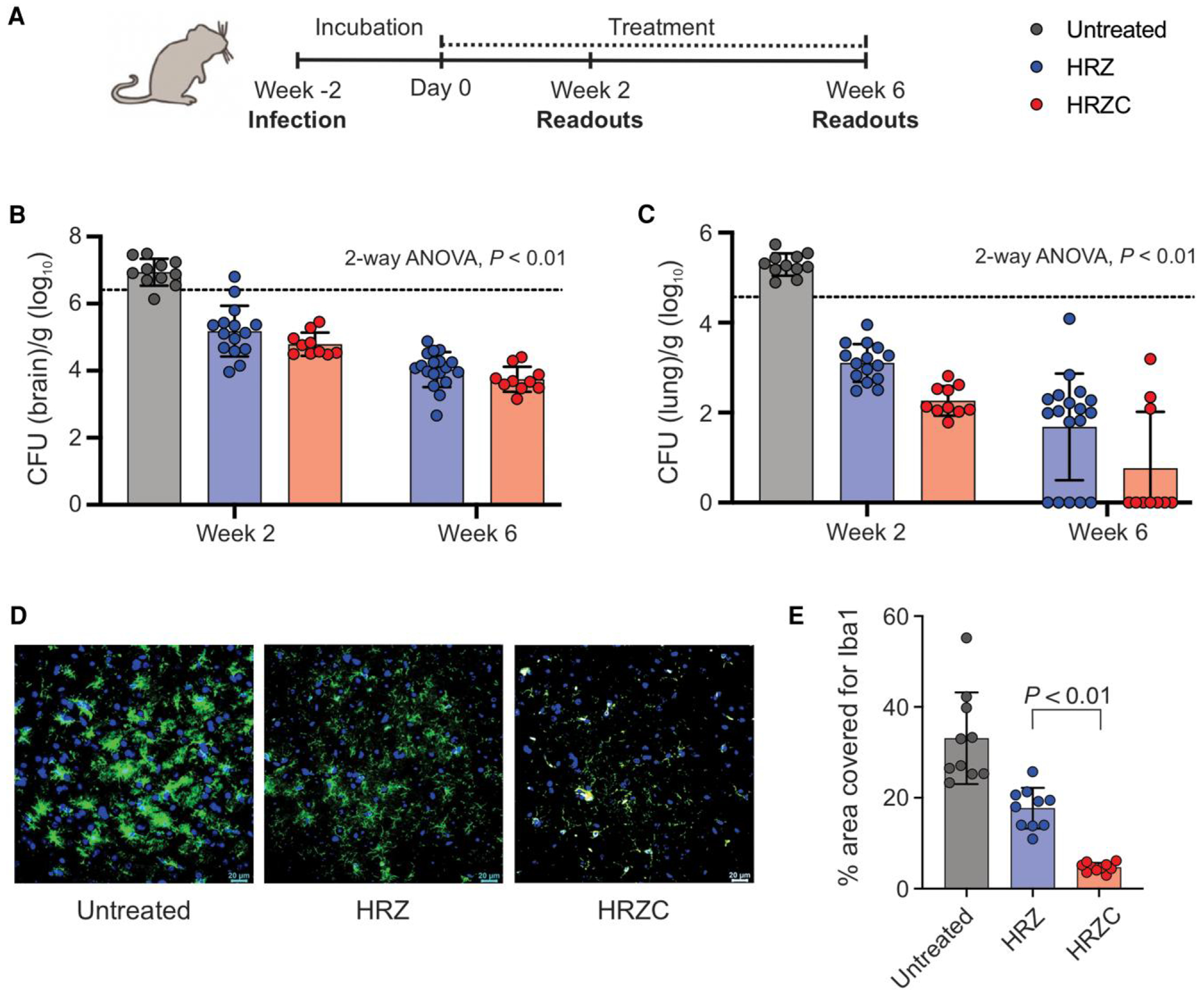
Treatment efficacy and neuroinflammation response of clofazimine-containing regimen. *A*, Experimental timeline for mouse TB meningitis model with infection at week −2, followed by treatment initiation at week 0 and readouts assessments after 2 and 6 weeks of treatment. *B*, Bacterial burden (CFU per gram brain, log_10_ scale) after 2 and 6 weeks of treatment with standard regimen (HRZ), and HRZ plus clofazimine (HRZC). *C*, Bacterial burden (CFU per gram lung, log_10_ scale) after 2 and 6 weeks of treatment with standard regimen (HRZ), and HRZ plus clofazimine (HRZC). Each dot represents an individual mouse; mean ± SD shown. HRZ: isoniazid/rifampin/pyrazinamide; HRZC: HRZ plus clofazimine; Dashed lines indicate baseline bacterial burden at treatment start. *D*, Representative confocal images showing Iba1-positive microglia (green) and DAPI-stained nuclei (blue) in mouse brain sections in untreated control and after 2 week of treatment with HRZ, and HRZ plus clofazimine (HRZC). Scale bar: 20 μm. *E*, Quantification of Iba1-positive area coverage (%) in brain tissue for each group. HRZ: isoniazid/rifampin/pyrazinamide. Each bar represents an individual mouse, 10 images were analyzed per mouse, bars show median ± IQR. Bacterial burden was analyzed using a two-way ANOVA. No multiple comparisons correction was applied, as all factors had only two levels and the interaction was not significant.

Female C3HeB/FeJ mice (6–8 weeks old, Jackson Laboratory; n = 15–20 animals per group) were anesthetized and infected with titrated frozen stocks via a burr hole using a Hamilton syringe in a stereotaxic frame [3], and the infection was incubated for 2 weeks to allow establishment of CNS infection and TB meningitis. After the incubation period, antibiotic regimens were administered for 6 weeks, with treatment efficacy assessed through microbiological and histopathological endpoints during and at the end of the study (Figure 1A). Further details on the animal model are provided in the Supplementary Materials. All animals were housed in controlled light and temperature rooms without cross-ventilation in a biosafety level-3 facility. All protocols were approved by the Johns Hopkins University Biosafety, Radiation Safety, Animal Care and Use Committees (MO19M382).

### Antimicrobial Treatment

Drug stocks were prepared and administered 5 days a week orally. All drugs were purchased from MedChem and administered at clinically relevant dose that matches pharmacokinetic (PK)/pharmacodynamic (PD)-based exposure in humans (Supplementary Table 1) [2], via oral gavage in a volume of 0.2 mL. Dexamethasone was administered intraperitoneally at human equipotent dosing [3]. Bacterial burden was quantified in whole organs (brain, lung and spleen) as colony forming units (CFU) following 2 and 6 weeks of treatments using 7H11 plates supplemented with activated charcoal.

### Mass Spectrometry for Clofazimine Concentrations

Tissues, plasma and cerebrospinal fluid (CSF) were collected at plasma T_max_ (8 hours ± 30 minutes) for clofazimine in mice, 2 weeks after treatment initiation. Clofazimine concentrations were quantified using validated ultra-high-performance liquid chromatography and tandem mass spectrometry (LC-MS/MS) at the Infectious Diseases Pharmacokinetics Laboratory of the University of Florida. The lower limit of detection was 0.01 μg/ml. Additional methodological details are provided in the Supplementary Materials.

### Neuroinflammatory Markers

Fixed brain tissues collected from animals 2 weeks after treatment initiation underwent immunofluorescence for ionized calcium-binding adaptor molecule 1 (Iba1, Thermo Fisher, MA5-36257, 1:500), a validated marker of microglial activation in TB meningitis models [3], followed by secondary Alexa-Fluor 488 antibody (1:100) and DAPI mounting.

Plasma samples were collected from untreated and treated animals 2 weeks after treatment initiation, and glial fibrillary acidic protein (GFAP) levels, a translational serum biomarker of astrocytic injury released into blood upon CNS damage, was quantified using an ELISA kit (Thermo Fisher EEL098) and normalized to total protein. Additional methodological details are provided in the Supplementary Materials.

### Statistical Analysis

Data were analyzed using Prism 10.2.2 (GraphPad). Bacterial burden is represented on a logarithmic scale (base 10) as mean ± SD. Statistical analysis of the effect of treatment on bacterial burden reduction at different time points was performed using a two-way ANOVA. Correlations between CFU and plasma GFAP levels were evaluated using Spearman’s rank correlation coefficient. No additional per-group analyses were performed, as no statistically significant differences between the slopes were found. All other data are represented as median ± IQR and comparison were made using a two-tailed Mann–Whitney U test. *P* values ≤.05 were considered statistically significant.

## RESULTS

### Bactericidal Activity

Mice with experimentally induced TB meningitis were randomly allocated to receive first-line regimen HRZ (isoniazid/rifampin/pyrazinamide) with or without clofazimine (HRZC). All regimens were given orally, and doses were prepared according to their human equipotent dosing; dexamethasone (at human equipotent dosing) was administered intraperitoneally with all regimens. After 2 weeks of treatment, the standard regimen HRZ with and without clofazimine led to a substantial reduction in brain bacterial burden compared with the untreated control. The addition of clofazimine enhanced the activity of the standard TB regimen (HRZ), with the HRZC group demonstrating significantly lower brain bacterial burden compared with HRZ (*P* < .01) (Figure 1B, Supplementary Figure 1A). Lung bactericidal activity mirrored the CNS readouts, with HRZC group achieving substantial reductions over time (*P* < .01) (Figure 1C, Supplementary Figure 1B).

### Brain Tissue Inflammation and Injury

Immunofluorescence quantification of Iba1, a marker of microglial activation (Figure 1D), was used to assess brain inflammation in animals. Quantification of Iba1 (Figure 1E) confirmed that addition of clofazimine (HRZC) significantly decreased microglial activation compared with HRZ-treated animals (*P* < .01).

Plasma GFAP levels, a marker of astrocytic activation and a quantifiable biomarker of neuroinflammation and brain damage, were assessed (Supplementary Figure 2A). Untreated mice showed the highest GFAP levels, consistent with CNS injury. HRZ treatment reduced GFAP levels, but mice receiving the clofazimine-containing regimen (HRZC) had lower plasma GFAP compared with HRZ (*P* = .11). Importantly, bacterial burden (CFU) positively correlated with plasma GFAP levels (r = 0.60, 95% CI .24–.82, *P* < .01), suggesting reduced brain damage in the context of effective antibacterial treatment (Supplementary Figure 2B).

### Tissue-specific Accumulation of Clofazimine

**Figure 2.**
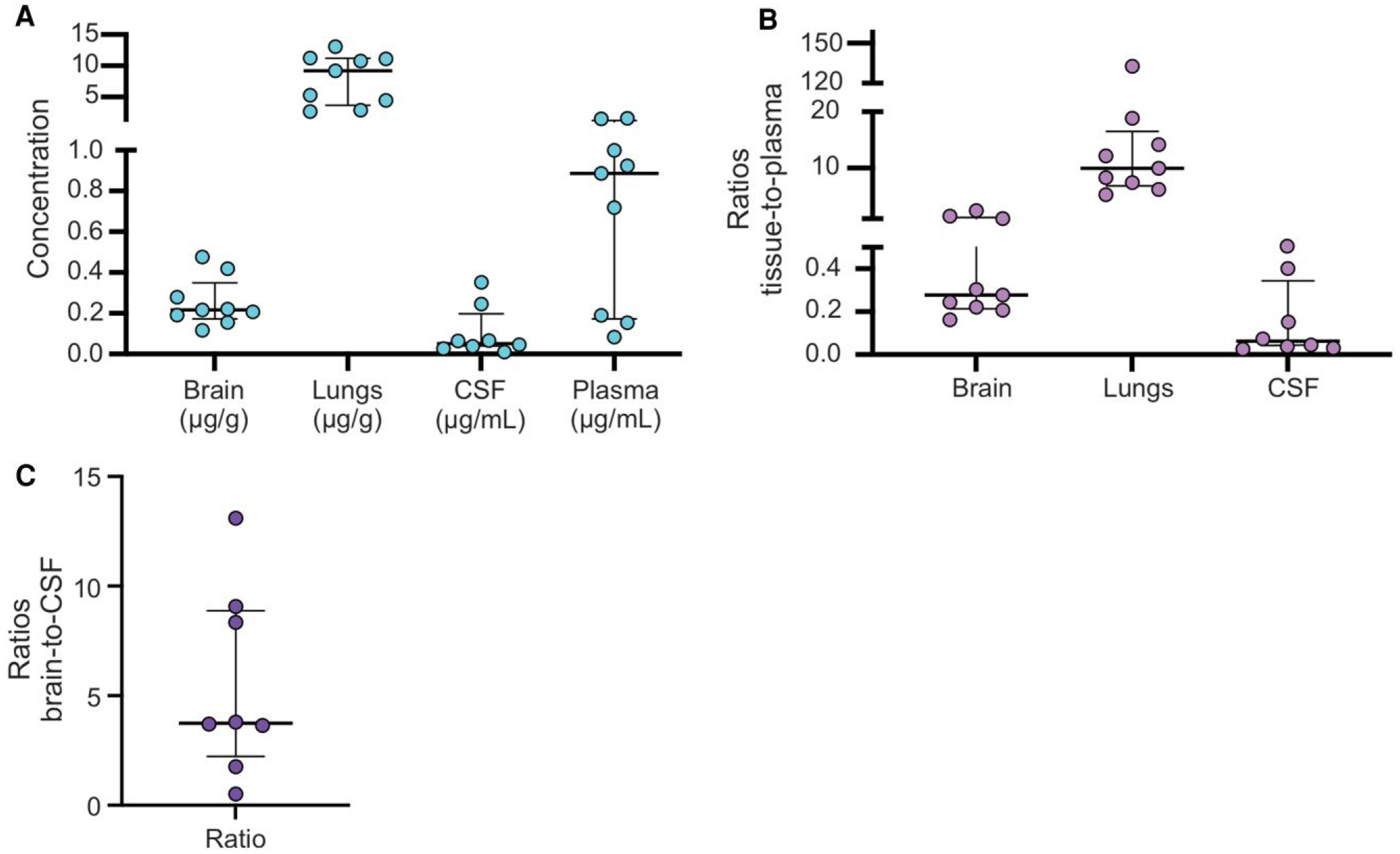
Biodistribution of clofazimine in TB meningitis infected mice. *A*, Absolute clofazimine concentrations measured by mass spectrometry in brain (μg/g), lungs (μg/g), cerebrospinal fluid (CSF, μg/mL), and plasma (μg/mL) at steady state 2 weeks into treatment. Each dot represents an individual animal; n = 9 animals; horizontal lines indicate median ± IQR. *B*, Tissue-to-plasma concentration ratios for clofazimine in brain, lung, and CSF. Each dot represents an individual mouse; median ± IQR shown. Note the relatively high lung and low CSF penetration compared with plasma, with moderate brain tissue accumulation. *C*, Brain-to-CSF concentrations ratios. Each dot represents an individual mouse; median ± IQR shown.

Clofazimine concentrations quantified after 2 weeks of treatment revealed distinct distribution patterns (Figure 2A). Median concentrations were highest in lung tissue (9.21 μg/g), followed by plasma (0.88 μg/mL), and much lower in the brain (0.22 μg/g) and CSF (0.05 μg/mL). Relative concentration ratios (tissue-to-plasma) (Figure 2B) confirmed predominant pulmonary accumulation of clofazimine, with lung-to-plasma ratios exceeding those for brain or CSF. Brain concentrations of clofazimine were substantially higher compared with CSF (*P* < .01), with a median brain-to-CSF ratio of 3.75 (IQR, 2.23–8.88) (Figure 2C).

## DISCUSSION

Clofazimine is emerging as a candidate for TB treatments owing to its favorable pharmacokinetics and dual antimycobacterial and immunomodulatory properties. Clofazimine-containing regimens, particularly for drug-resistant TB, have demonstrated enhanced antibacterial efficacy and noninferiority in shortened treatment regimens compared with standard of care in previous preclinical [2] and clinical studies [4, 5]. Notably, the recent Clo-Fast trial results demonstrate that a 3-month clofazimine-rifapentine regimen was noninferior to the standard 6-month regimen for drug-susceptible TB [6]. Although the 3-month regimen had a higher relapse rate, the clofazimine-containing regimen achieved slightly earlier culture conversions compared with the control arm, despite enrolling a higher proportion of patients with higher bacillary burden (AFB smear grade 3). Evidence supporting the efficacy of clofazimine in CNS TB remains limited, given the minimal concentrations achievable in CSF of patients [7]. Our study addresses key gaps demonstrating that clofazimine achieves measurable concentrations in the CNS and enhances the activity of the standard TB treatment in the brain as well as the lungs—a critical benefit given the dual CNS and pulmonary involvement often seen in patients with TB meningitis. The improved outcomes with the isoniazid-rifampin-pyrazinamide-clofazimine (HRZC) regimen may reflect the distinct mechanism of action for clofazimine and tissue accumulation [8]. This is consistent with evidence that clofazimine enhances intracellular killing through membrane disruption and reactive oxygen species generation [9], complementing the effect of first-line antibiotics against *M. tuberculosis*. We utilized mouse dosing of 6.25 mg/kg/day comparable to the 100 mg/day dose in human dose [2, 6]. However, a higher clofazimine dose, could further increase the activity of the regimen.

Although CSF drug concentrations were low and inconsistent (<0.1 ug/mL), brain tissue concentrations of clofazimine exceeded the minimum inhibitory concentration for *M. tuberculosis* [10]. This finding is consistent with measurable intracerebral distribution of clofazimine in a prior animal study [8], as well as with clinical studies suggesting low or undetectable CSF concentrations of clofazimine in patients with pulmonary TB or TB meningitis [7, 11]. The low CSF-to-plasma ratio reflects the high-protein binding, as well as the lipophilicity and slow equilibration half-life of clofazimine. Since only the protein-unbound drug would be available for achieving a plasma-to-CSF equilibrium, isolated quantification of clofazimine in the CSF may underestimate CNS penetration [11, 12]. Of note, brain and CSF reflect distinct compartments and although our mouse CSF data are consistent with clinical studies demonstrating low clofazimine levels in the CSF, human brain exposures may be different from those observed in the mouse studies.

Addition of clofazimine to the standard TB regimen also conferred neuroprotection, evidenced by lower serum GFAP levels in mice receiving clofazimine in addition to the standard TB treatment. Elevated GFAP reflects astrocytic activation and brain tissue damage in response to infection or injury [13]. Immunofluorescence findings of reduced microglial activation (Iba1) further corroborate these results [14]. However, in this study the apparent anti-inflammatory effects cannot be fully disentangled from the reductions in bacterial burden, and while clofazimine may also directly affect host responses and dampen inflammation [15], the relative contribution of these effects is uncertain.

In conclusion, these data support clofazimine as a rational adjunct to be incorporated into TB regimens, ensuring CNS penetration while combining potent intracerebral bactericidal activity with measurable attenuation of neuroinflammation and brain damage. Emerging clinical data demonstrating the efficacy and safety of clofazimine for pulmonary TB support further investigation and clinical evaluation of clofazimine-based regimens for TB meningitis.

## Supplementary Material

Supplementary data

Supplementary materials are available at The Journal of Infectious Diseases online (http://jid.oxfordjournals.org/). Supplementary materials consist of data provided by the author that are published to benefit the reader. The posted materials are not copyedited. The contents of all supplementary data are the sole responsibility of the authors. Questions or messages regarding errors should be addressed to the author.

## Data Availability

All data from this study are present in the paper and/or the Supplementary Materials

## References

[1] Jain SK, Tobin DM, Tucker EW, Tuberculous meningitis: a roadmap for advancing basic and translational research. Nat Immunol 2018; 19:521–5. 29777209 10.1038/s41590-018-0119-x PMC6089350

[2] Swanson RV, Adamson J, Moodley C, Pharmacokinetics and pharmacodynamics of clofazimine in a mouse model of tuberculosis. Antimicrob Agents Chemother 2015; 59:3042–51. 25753644 10.1128/AAC.00260-15 PMC4432183

[3] Ruiz-Bedoya CA, Mota F, Tucker EW, High-dose rifampin improves bactericidal activity without increased intracerebral inflammation in animal models of tuberculous meningitis. J Clin Invest 2022; 132:e155851. 35085105 10.1172/JCI155851 PMC8920328

[4] Nyang’wa BT, Berry C, Kazounis E, A 24-week, all-oral regimen for rifampin-resistant tuberculosis. N Engl J Med 2022; 387:2331–43. 36546625 10.1056/NEJMoa2117166

[5] Shafiq N, Kumar A, Vohra V, Four-month clofazimine regimen for susceptible pulmonary TB: a randomized clinical trial. J Antimicrob Chemother 2025; 80:2100–8. 40478219 10.1093/jac/dkaf176

[6] Metcalfe JZ, Weir IR, Scarsi KK, A 3-month clofazimine-rifapentine-containing regimen for drug-susceptible tuberculosis versus standard of care (Clo-Fast): a randomised, open-label, phase 2c clinical trial. Lancet Infect Dis 2026; 26:46–54. 40915311 10.1016/S1473-3099(25)00436-0 PMC12990188

[7] Kempker RR, Smith AGC, Avaliani T, Cycloserine and linezolid for Tuberculosis meningitis: pharmacokinetic evidence of potential usefulness. Clin Infect Dis 2022; 75:682–9. 34849645 10.1093/cid/ciab992 PMC9464073

[8] Baijnath S, Naiker S, Shobo A, Evidence for the presence of clofazimine and its distribution in the healthy mouse brain. J Mol Histol 2015; 46:439–42. 26208572 10.1007/s10735-015-9634-3

[9] Arbiser JL, Moschella SL. Clofazimine: a review of its medical uses and mechanisms of action. J Am Acad Dermatol 1995; 32:241–7. 7829710 10.1016/0190-9622(95)90134-5

[10] Park S, Jung J, Kim J, Han SB, Ryoo S. Investigation of clofazimine resistance and genetic mutations in drug-resistant Mycobacterium Tuberculosis isolates. J Clin Med 2022; 11: 1927. 35407536 10.3390/jcm11071927 PMC9000149

[11] Upton CM, Calderin JM, Diacon AH, Cerebrospinal fluid penetration of cycloserine/terizidone and clofazimine in patients with pulmonary TB. Antimicrob Agents Chemother 2025; 69:e0093125. 41118340 10.1128/aac.00931-25 PMC12691663

[12] Baik J, Stringer KA, Mane G, Rosania GR. Multiscale distribution and bioaccumulation analysis of clofazimine reveals a massive immune system-mediated xenobiotic sequestration response. Antimicrob Agents Chemother 2013; 57:1218–30. 23263006 10.1128/AAC.01731-12 PMC3591914

[13] Giovannoni F, Quintana FJ. The role of astrocytes in CNS inflammation. Trends Immunol 2020; 41:805–19. 32800705 10.1016/j.it.2020.07.007 PMC8284746

[14] Li X, Luo X, Wang B, Fu L, Chen X, Lu Y. Clofazimine inhibits innate immunity against Mycobacterium tuberculosis by NF-kappaB. mSphere 2024; 9:e0025424. 39046230 10.1128/msphere.00254-24 PMC11351037

[15] Xu J, Koval A, Katanaev VL. Clofazimine: a journey of a drug. Biomed Pharmacother 2023; 167:115539. 37742606 10.1016/j.biopha.2023.115539

