# Supplementary material for: Addition of Clofazimine Enhances the Activity of Standard Treatment Regimen in a Mouse Model of Tuberculous Meningitis
Source: J Infect Dis. 2026 Feb 23;234(1):e195–9. doi: 10.1093/infdis/jiag123 (PMC13094629; doi:10.1093/infdis/jiag123)
Supplement: jiag123_Supplementary_Data [file jiag123_supplementary_data.docx]

**Addition of Clofazimine Enhances the Activity of Standard Treatment Regimen in a Mouse Model of Tuberculous Meningitis**

Supplementary Information

**METHODS**

**Model establishment and experimental design**

Female C3HeB/FeJ mice (6-8 weeks old, Jackson Laboratory) were anesthetized and infected with frozen titrated stocks of *Mycobacterium tuberculosis* H37Rv via stereotaxic intracerebral injection (burr hole using Micro-Drill Kit, Braintree Scientific Inc.; Hamilton syringe #88000; David Kopf model 900 stereotaxic frame; coordinates: 0.6 mm dorsal to bregma, 1.2 mm lateral to midline, 2 mm ventral), as described previously [1-3]. Treatments were administered 2-weeks after the infection, for 6 weeks (5 days/week). Clofazimine concentrations were quantified using validated ultra-high-performance liquid chromatography (UPLC) and tandem mass spectrometry (LC-MS/MS) at the Infectious Diseases Pharmacokinetics Laboratory of the University of Florida [4].

**Iba1 immunofluorescence for microglial activation**

Ionized calcium-binding adaptor molecule 1 (Iba1) is a microglia-specific calcium-binding protein widely used as a marker for microglial activation and CNS macrophage infiltration in preclinical models of neuroinflammation, including TB meningitis. Upon activation, Iba1-expressing cells undergo morphologic changes (e.g., amoeboid hypertrophy) and increased staining intensity, reflecting pro-inflammatory states in response to infection or injury. In TB meningitis mouse and rabbit models, Iba1 immunofluorescence has been validated to quantify meningeal and parenchymal microgliosis correlating with bacterial burden and pathology [5, 6]. Here, % area staining was quantified in 10 fields per brain section using FIJI ImageJ after 40x imaging (Leica DM6B), providing a sensitive histologic readout of treatment effects on neuroinflammation.

**Plasma GFAP ELISA for astrocytic injury**

Glial fibrillary acidic protein (GFAP) is an astrocyte intermediate filament released into plasma upon reactive astrogliosis or cellular injury, serving as a quantifiable serum biomarker of CNS damage. Plasma GFAP elevations parallel brain injury severity in mouse models of trauma, neurodegeneration, and infection, with sensitive immunoassays enabling non-invasive monitoring [7]. Translationally, serum/plasma GFAP is FDA-cleared for traumatic brain injury diagnosis and is prognostic across human CNS disorders (e.g., stroke, Alzheimer's, infections) [8]. In this study, GFAP was quantified in plasma (week 2) using a validated ELISA kit (Thermo Fisher Scientific, EEL098), normalized to total protein, to assess the impact of clofazimine on astrocytic damage, a key driver of TBM sequelae.

**FIGURES**

**
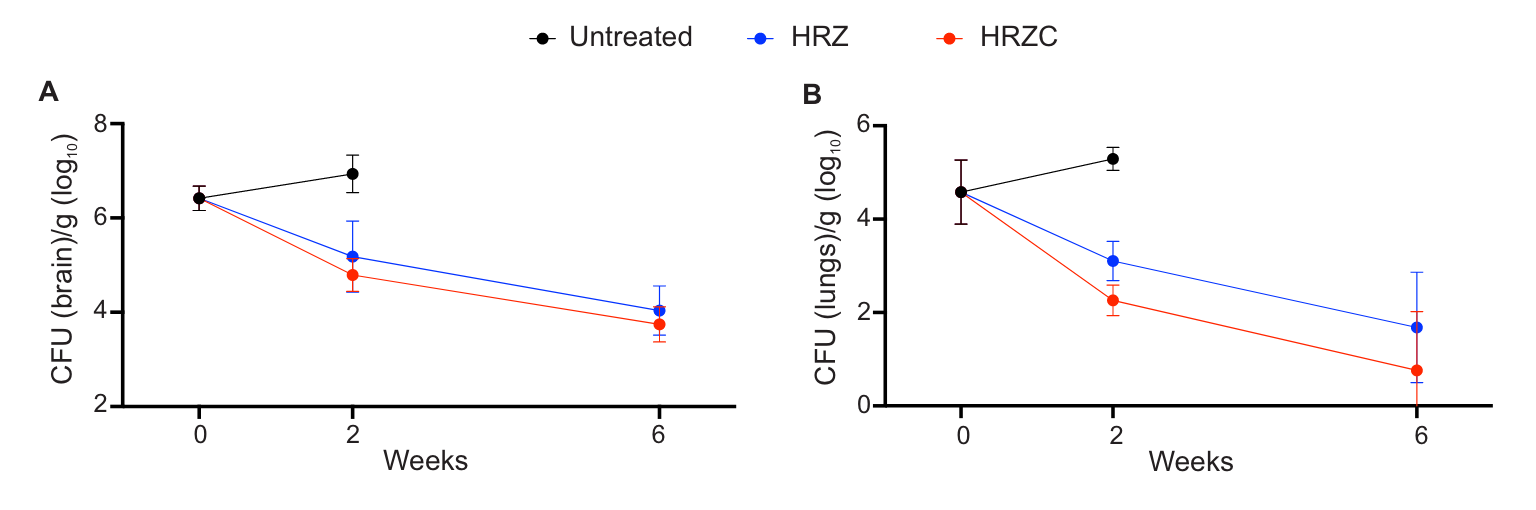
**

**Figure S1. Efficacy of clofazimine-containing regimens.** (**A**) Longitudinal changes in brain CFU (log₁₀) over the course of treatment in the controls and treatment regimens, mean ± SD shown. (**B**) Longitudinal changes in lung CFU (log₁₀) over the course of treatment in the control and treatment regimens; n = 10-15 animals per timepoint per group; mean ± SD shown. HRZ: isoniazid/rifampin/pyrazinamide; HRZC: HRZ plus clofazimine.


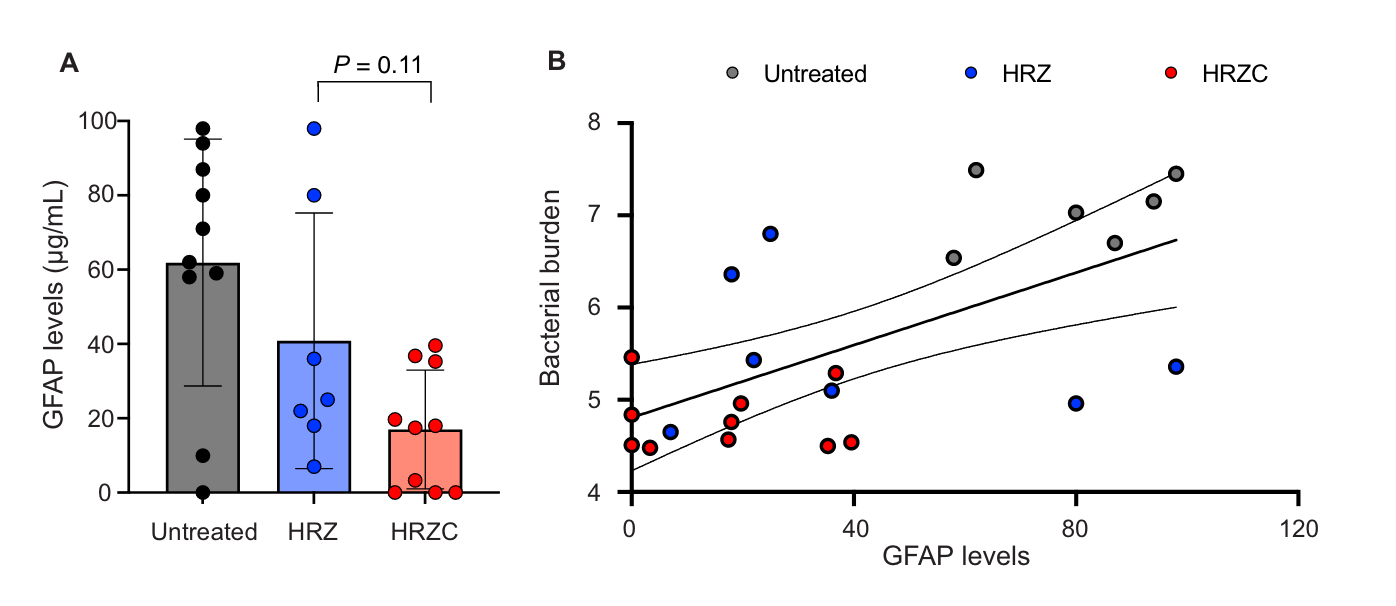


**Figure S2. Plasma GFAP concentrations and correlation.** (**A**) Plasma levels of glial fibrillary acidic protein (GFAP, µg/mL) were assessed in mice with TB meningitis after 2 weeks of treatment with standard therapy (HRZ), HRZ plus clofazimine (HRZC), and untreated controls. All treatment groups demonstrated lower GFAP concentrations relative to the untreated control group, with the greatest reduction observed in the HRZC regimen. (**B**) Correlation between plasma GFAP levels (µg/mL) and bacterial burden (CFU per gram brain, log₁₀ scale). The solid black line represents the pooled linear correlation across all groups. A significant positive correlation was observed between plasma GFAP levels and CFU (Spearman’s r = 0.60, 95% CI 0.24 - 0.82, *P* < 0.01). HRZ: isoniazid/rifampin/pyrazinamide. Each dot represents an individual mouse; n = 7-10 animals per group; bars show median ± IQR. Statistical comparison was made using the Mann-Whitney U two-tailed test for panel A.

**TABLES**

**Table S1.** Mouse doses and their corresponding human equipotent dose for tuberculosis treatment regimens

| **Drug** | **Mouse dose** | **Human dose** |
| --- | --- | --- |
| Isoniazid | 10 mg/kg | 10 mg/kg |
| Pyrazinamide | 150 mg/kg | 25 mg/kg |
| Rifampin | 10 mg/kg | 10 mg/kg |
| Clofazimine | 6.25 mg/kg/day [9] | 100 mg [10] |
| Dexamethasone | 2 mg/kg/day | 0.4 mg/kg |

**REFERENCES**

1. Ruiz-Bedoya CA, Mota F, Tucker EW, et al. High-dose rifampin improves bactericidal activity without increased intracerebral inflammation in animal models of tuberculous meningitis. J Clin Invest **2022**; 132.

2. Chen X, Arun B, Nino-Meza OJ, et al. Dynamic PET reveals compartmentalized brain and lung tissue antibiotic exposures of tuberculosis drugs. Nat Commun **2024**; 15:6657.

3. Mota F, Ruiz-Bedoya CA, Tucker EW, et al. Dynamic (18)F-Pretomanid PET imaging in animal models of TB meningitis and human studies. Nat Commun **2022**; 13:7974.

4. Alghamdi WA, Al-Shaer MH, Kipiani M, et al. Pharmacokinetics of bedaquiline, delamanid and clofazimine in patients with multidrug-resistant tuberculosis. J Antimicrob Chemother **2021**; 76:1019-24.

5. Latham AS, Geer CE, Ackart DF, et al. Gliosis, misfolded protein aggregation, and neuronal loss in a guinea pig model of pulmonary tuberculosis. Front Neurosci **2023**; 17:1157652.

6. Tucker EW, Pokkali S, Zhang Z, et al. Microglia activation in a pediatric rabbit model of tuberculous meningitis. Dis Model Mech **2016**; 9:1497-506.

7. Kmetova K, Drobna D, Liptak R, Hodosy J, Celec P. Early dynamics of glial fibrillary acidic protein and extracellular DNA in plasma of mice after closed head traumatic brain injury. Neurochirurgie **2022**; 68:e68-e74.

8. Korley FK, Jain S, Sun X, et al. Prognostic value of day-of-injury plasma GFAP and UCH-L1 concentrations for predicting functional recovery after traumatic brain injury in patients from the US TRACK-TBI cohort: an observational cohort study. Lancet Neurol **2022**; 21:803-13.

9. Swanson RV, Adamson J, Moodley C, et al. Pharmacokinetics and pharmacodynamics of clofazimine in a mouse model of tuberculosis. Antimicrob Agents Chemother **2015**; 59:3042-51.

10. Metcalfe JZ, Weir IR, Scarsi KK, et al. A 3-month clofazimine-rifapentine-containing regimen for drug-susceptible tuberculosis versus standard of care (Clo-Fast): a randomised, open-label, phase 2c clinical trial. Lancet Infect Dis **2026**; 26:46-54.
